# MiRNA expression deregulation correlates with the Oncotype DX^®^ DCIS score

**DOI:** 10.1186/s13058-022-01558-4

**Published:** 2022-09-12

**Authors:** Olivier Loudig, Megan I. Mitchell, Iddo Z. Ben-Dov, Christina Liu, Susan Fineberg

**Affiliations:** 1 Center for Discovery and Innovation, Hackensack Meridian Health, Nutley, NJ 07110 USA; 2 Department of Nephrology and Hypertension, Hadassah Medical Center, 91120 Jerusalem, Israel; 3 Department of Pathology, Montefiore Medical Center and the Albert Einstein College of Medicine, Bronx, NY 10461 USA

## Abstract

**Background:**

Current clinical criteria do not discriminate well between women who will or those who will not develop ipsilateral invasive breast cancer (IBC), or a DCIS recurrence after a ductal carcinoma in situ (DCIS) diagnosis. The 12-gene Oncotype DX® DCIS assay (RT qPCR gene-based scoring system) was established and shown to predict the risk of subsequent ipsilateral IBC or DCIS recurrence. Recent studies have shown that microRNA (miRNA) expression deregulation can contribute to the development of IBC, but very few have evaluated miRNA deregulation in DCIS lesions. In this study, we sought to determine whether specific miRNA expression changes may correlate with Oncotype DX® DCIS scores.

**Methods:**

For this study, we used archived formalin-fixed, paraffin-embedded (FFPE) specimens from 41 women diagnosed with DCIS between 2012 and 2018. The DCIS lesions were stratified into low (*n* = 26), intermediate (*n* = 10), and high (*n* = 5) risk score groups using the Oncotype DX® DCIS assay. Total RNA was extracted from DCIS lesions by macro-dissection of unstained FFPE sections, and next-generation small-RNA sequencing was performed. We evaluated the correlation between miRNA expression data and Oncotype score, as well as patient age. RT-qPCR validations were performed to validate the topmost differentially expressed miRNAs identified between the different risk score groups.

**Results:**

MiRNA sequencing of 32 FFPE DCIS specimens from the three different risk group scores identified a correlation between expression deregulation of 17 miRNAs and Oncotype scores. Our analyses also revealed a correlation between the expression deregulation of 9 miRNAs and the patient’s age. Based on these results, a total of 15 miRNAs were selected for RT-qPCR validation. Of these, miR-190b (*p* = 0.043), miR-135a (*p* = 0.05), miR-205 (*p* = 0.00056), miR-30c (*p* = 0.011), and miR-744 (*p* = 0.038) showed a decreased expression in the intermediate/high Oncotype group when compared to the low-risk score group. A composite risk score was established using these 5 miRNAs and indicated a significant association between miRNA expression deregulation and the Oncotype DX® DCIS Score (*p* < 0.0021), between high/intermediate and low risk groups.

**Conclusions:**

Our analyses identified a subset of 5 miRNAs able to discriminate between Oncotype DX® DCIS score subgroups. Together, our data suggest that miRNA expression analysis may add value to the predictive and prognostic evaluation of DCIS lesions.

**Supplementary Information:**

The online version contains supplementary material available at 10.1186/s13058-022-01558-4.

## Introduction

Ductal carcinoma *in situ* (DCIS) of the breast refers to a premalignant lesion composed of malignant epithelial cells that have yet to invade through the basement membrane of the duct into the surrounding stroma [1–3]. This heterogeneous group of neoplastic lesions, which is considered a precursor to invasive ductal carcinoma (IDC), accounts for approximately 20% of newly diagnosed breast cancer cases in the USA [4] with incidence peaking in women aged 65–69 years. Clinically, DCIS is commonly detected through routine screening mammograms, and often presents as microcalcifications (approximately 80–90% of DCIS cases) [5] and less often as a palpable mass (approximately 8% of cases) [6, 7]. Advancements in screening mammography coupled with additional diagnostic mammography, ultrasound, and/or breast MRI for patients who present with a suspicious lesion are not only important for the characterization of ipsilateral disease [8–10] but have also greatly increased the detection of DCIS lesions [11]. As a result, significant increases in DCIS incidence have been observed across all ages but more predominantly in postmenopausal women [12]. Indeed, data from the surveillance, epidemiology, and end results (SEER) program have shown that the incidence of DCIS increased approximately sevenfold between 1980 and 2001 [11].

DCIS is considered a non-obligatory precursor of invasive breast carcinoma (IBC), where left untreated 14–64% of DCIS patients progress to invasive cancer within 10 years [13–15]. It has been estimated that women with a history of DCIS have a 1.5–10-fold greater risk of subsequent IBC, and thus women diagnosed with DCIS have a threefold greater risk of dying from breast cancer than those without [16, 17]. DCIS is stratified into three grades based on nuclear grade, i.e., corresponding to low (grade 1), intermediate (grade 2), and high grade (grade 3) [18]. Although not all cases of DCIS will progress to invasive breast cancer it is thought that the grade of DCIS at the time of diagnosis is related to the likelihood of progression to IBC and the rapidity with which it is likely to occur [19]. Several meta-analysis studies suggest that an increased risk of ipsilateral recurrence is associated with high-grade DCIS; however, a meta-analysis by Zhang et al. suggests that DCIS grade is not significantly associated with the risk of invasive recurrence [20]. Inconsistencies in the ability to predict progression to invasive breast disease for those DCIS patients indicate that nuclear grade is not an ideal discriminant. The fact that among women with DCIS treated with surgical resection alone 20–40% of cases experience a recurrence in the same breast, with half of those women recurring with IBC [21, 22] further highlights the need for identification of consistent and reproducible predictors of DCIS outcomes.

Currently, the majority of women diagnosed with DCIS are treated with breast-conserving surgery followed by radiation therapy (RT). Although, the addition of radiation therapy decreases local recurrence by about 50%, from 28.1 to 12.9%, the 15-year breast cancer-specific mortality rate from DCIS treated by lumpectomy alone without radiation therapy is only 2.33% [23, 24]. Consequently, a majority of patients diagnosed with DCIS lesions undergo excessive treatments, and thus improved diagnostic tools are urgently needed to predict both the risk of local recurrence and/or DCIS progression to IBC. In addition to DCIS grade, several clinicopathological features of DCIS routinely assessed in all cases have emerged for their association with higher local recurrence rates and include absence of estrogen receptor, larger DCIS lesion size, positive margin status, and younger patient age [25]. Additional immunohistochemical markers which are not routinely assessed during initial DCIS diagnosis have shown an association with progression to IBC and include expression of p16, COX2 and Ki67 and HER2 [26].

In 2012, the Oncotype DX^®^ Breast DCIS test was developed for evaluation of DCIS lesions by RT-qPCR quantification of 12 of the 21 genes from the original Oncotype DX^®^ Breast cancer assay, utilized for recurrence risk stratification of primary breast tumors [27]. This 12-gene assay is used to obtain a score based on the expression of 7 cancer-related genes (Ki67, AURKA/STK15, BIRC5/survivin, CCNB1, MYBL2, PGR, and GSTM1) and 5 normalizing reference genes (ACTB, GAPDH, RPLPO, GUS, and TFRC) [27]. Expression analysis can be done on either whole tissue [28–30], or archived formalin-fixed paraffin-embedded (FFPE) sections [31–33]. Clinically tested, the Oncotype DX^®^ Breast DCIS assay has been used to predict the 10-year local recurrence risk of ipsilateral DCIS and/or IBC recurrence by stratifying patients into low (< 39), intermediate (39–54), and high (>= 55) risk score groups [34]. When originally introduced in 2012, the recurrence risk was estimated solely based on the DCIS score; however, in 2018 it was refined to include patient age in local recurrence risk estimates; specifically, premenopausal patients were reported at higher local recurrence risk at all DCIS scores [35, 36]. This molecular scoring system has been moderately used by clinicians to evaluate the risk of recurrence as well as for the evaluation of treatment strategies.

MicroRNAs (miRNA) are a class of endogenous short (22±2 nucleotides), noncoding RNAs that are known to be involved in virtually all cellular processes [37]. MiRNAs are key posttranscriptional regulators of gene expression which generally exert their effects by binding directly to the 3’ untranslated region (UTR) of their target mRNA transcripts, destabilizing the mRNA, leading to translational silencing and ultimately the repression of protein production [38–40]. Their expression regulation effects are intricate, as a single miRNA can regulate the expression of multiple genes [41], and each mRNA may be regulated by multiple miRNAs [42]. Tissue miRNA expression studies have examined their expression changes in relation to different physiological and pathological states [43]. Indeed, the deregulation of miRNA expression has been shown to contribute to the development and progression of a variety of human malignancies, including breast cancer [44], categorizing miRNAs in a novel class of oncogenes and/or tumor suppressor RNA molecules [45].

Studies that we have performed using archived FFPE tissue specimens have demonstrated that optimized high-throughput gene expression technologies are suitable for the retrospective analysis of miRNAs [46–49]. Our analyses with FFPE tissues as old as 35 years have shown reproducible detection of miRNAs highlighting their robust nature and molecular resilience to degradation and decay [48]. Recently, our laboratory identified a subset of miRNAs, which displayed differential expression between DCIS lesions of patients who went on to develop invasive breast cancer, by comparison to DCIS lesions from control patients who did not develop breast cancer within the same time interval [48].

In this study, we selected archived FFPE specimens from 41 women diagnosed with DCIS between 2012 and 2018, whose DCIS lesions were initially classified by the Oncotype DX^®^ Breast DCIS score, and we evaluated the correlation between miRNA expression and DCIS scores. We used next-generation small-RNA sequencing to initially identify miRNA expression differences and then used RT quantitative PCR to validate our findings. As an additional measure, we evaluated miRNA expression changes based on the age of the patients diagnosed with DCIS.

## Materials and methods

### Clinical specimen selection and processing

We identified archived formalin-fixed paraffin-embedded specimens from 41 women diagnosed with ductal carcinoma in situ (DCIS) between 2012 and 2018, whose DCIS lesions underwent evaluation with the Oncotype DX® Breast DCIS Score test. Specimens were identified from the breast cancer database of the Montefiore Medical Center (Bronx, NY). We selected samples representing low (< 39; *n* = 26), intermediate (39–54, *n* = 11), and high (>= 55, *n* = 5) Oncotype DX® breast DCIS score groups. The FFPE blocks used for Oncotype testing were retrieved from the Department of Pathology, and one 5 mm Hematoxylin and Eosin (H&E) section was obtained and reviewed by the study breast pathologist for localization of DCIS lesions. These H&E sections were used for microscope-guided macro-dissection of the DCIS lesions from sequentially sectioned, unstained 10 µm sections (*n* = 10 sections per case). Macro-dissected lesions were transferred into siliconized Eppendorf tubes for subsequent RNA extraction. Prior to study initiation, proper IRB approval was obtained from Montefiore Medical Center (IRB # 2014-3292) and from Hackensack University Medical Center (IRB # 2018-0156).

### RNA extraction and quantification

RNA was extracted as previously described by Loudig et al. [48]. Briefly, macro-dissected tissue lesions underwent a series of CitriSolv, 100% ethanol, and 95% ethanol washes to remove paraffin. This was followed by a 12,000 RPM centrifugation step to collect the tissue. PBS with RNase inhibitors was added for sample rehydration prior to Proteinase K (3 mg/ml) digestion for 1 h at 59 °C. The individually digested clinical specimens were subjected to 1-Butanol (ThermoFisher, #A399-1) concentration before undergoing TRIzol RNA extraction using a protocol described by Kotorashvili et al. [50]. Following the addition of Chloroform and 12,000 g centrifugation for 5 min at 4 °C, RNA was recovered from the upper aqueous phase. Samples were then stored at − 80 °C until total RNA was precipitated via incubation with linear acrylamide (0.1 mg/µl), sodium acetate (3 M), and 100% ethanol overnight. The next day, total RNA was pelleted by centrifugation at 14,000 RPM for 30 min at 4 °C. Total RNA was resuspended in 1 × TE buffer, incubated at 70 °C for 30 min for cross-linkage removal, and subsequently quantified using a NanoDrop ND-200. Samples were analyzed on an Agilent Bioanalyzer total RNA chip for the accurate evaluation of RNA concentration and quality across all DCIS specimens. Total RNA was then divided into 100 ng aliquots for small-RNA sequencing library preparation and 2 ng/µl aliquots for qPCR analyses.

### miRNA expression profiling using next-generation sequencing

Small-RNA sequencing from FFPE samples was performed using the cDNA library preparation protocol as described by Loudig et al. [48]. Briefly, 18 ligations were individually prepared using different 3’ adenylated barcode adapters (1 µl of 50 µM), where each run containing 18 samples (100 ng of total RNA each) was established. A master mix containing 0.026 nM of a custom calibrator cocktail was prepared, and 8.5 µl of this master-mix was added to each sample (9.5 µl) followed by the addition of 1 µl of 50 µM 3’ barcode adapter and incubation with, truncated K227Q T4 RNA Ligase 2 (New England Biolabs, #M0351L) at 4 °C overnight. The next day, samples were heated at 90 °C to inactivate the enzyme and individual precipitations were performed by the addition of 1.2 µl GlycoBlue/NaCl mix (1 µl GlycoBlue™ Co-precipitant (15 mg/ml; ThermoFisher, #AM9516) / 26 µl 5 M NaCl (ThermoFisher, #AM9579)) and 63 µl of 100% ethanol, samples were then combined into a single tube and placed on ice for 1 h followed by centrifugation for 1 h at 15,000 RPM, at 4 °C. The combined library pellet was vacuum-dried, resuspended in nuclease-free water and denaturing PAA gel loading dye, and run on a 15% urea-PAGE gel alongside oligonucleotide size markers (19 nt and 24 nt, IDT) for visual size reference to guide the excision of the 3’ ligated RNA product. Excised gel pieces were placed into gel breaker tubes (IST Engineering, #3388-100) and subjected to crushing by means of centrifugation and incubation in 400 mM NaCl solution with agitation (1,100 RPM) overnight. The next day, the RNA pellet was again subjected to filtration and precipitation with 100% ethanol on ice for 1 h followed by centrifugation at 15,000 RPM for 1 h. Samples were then ligated to a 5’ adapter using T4 RNA Ligase 1 (New England Biolabs, #M0204L) for 1 h at 37 °C and separated on a 12% urea-PAGE gel, with 5’ ligated size markers for visual size reference. Excised gel fragments were subjected to crushing in gel breaker tubes and incubated in a 300 mM NaCl solution containing 1 µl of 3’ PCR primer (100 µM), and incubated overnight on a thermomixer set to 4 °C and 1,100 RPM. Following filtration, precipitation on ice, and centrifugation at 15,000 RPM for 1 h the RNA pellet, resuspended in nuclease-free water was subjected to reverse transcription using SuperScript III (ThermoFisher, #18080-093) at 50 °C for 30 min. An initial pilot PCR was performed, where 12 µl aliquots were withdrawn at cycles 10, 12, 14, 16, 18, 20, and 22 and run on a 2.5% agarose gel for visualization and selection of cycle number which yielded optimal target-to-primer dimer ratio. A large-scale PCR (six PCRs) was then set up, subjected to the optimal number of amplification cycles identified in the pilot, 10 µl of each reaction was visualized on a 2.5% agarose gel, combined, and then precipitated overnight at − 20 °C. The resultant PCR product was then subjected to PmeI digestion for removal of size markers and run on a 2.5% agarose gel. The resultant 100 nt PCR library product was then excised and purified using a QIAquick gel extraction kit (Qiagen, #28704). Quantification was done using the Qubit dsDNA HS kit (ThermoFisher, #Q32854), and libraries were then sequenced (single-read 50 cycles) on a HiSeq 2500 Sequencing System (Illumina, #SY-401-2501). Raw sequencing data files (FASTQ) were processed for adapter trimming and small-RNA alignment to the hg19 genome and small-RNA databases. Read counts were normalized to total counts and subjected to statistical analyses.

### TaqMan reverse transcription and qPCR analyses

Target miRNA expression quantification was performed in triplicate, and results were normalized to the endogenous control, RNU6B. Briefly, 10 ng of total RNA from the FFPE clinical specimens was reverse transcribed per miRNA using TaqMan microRNA assays (ThermoFisher, #4427975), and TaqMan® microRNA master mix PCR kits, following manufacturer’s instructions. For individual transcript quantification, 4.36 ul of cDNA was used to set up the three individual qPCR experiments. TaqMan miRNA primer assays selected for these qPCR validations included hsa-miR-19a (#000395), hsa-miR-19b (#000396), hsa-miR-30c (#000419), hsa-miR-132 (#000457), hsa-miR-135a (#000460), hsa-miR-135b (#002261), hsa-miR-142-3p (#000464), hsa-miR-142-5p (#002248), hsa-miR-146a (#000468), hsa-miR-150 (#000473) hsa-miR-155 (#002623), hsa-miR-190b (#002263), hsa-miR-193b (#002367), hsa-miR-205 (#000509), hsa-miR-744 (#002324), and RNU6B (#001093). Reactions were set up in microAmp fast optical 96-well reaction plates (ThermoFisher, #4346906) and sealed with microAmp optical adhesive film (ThermoFisher, #4311971). qPCR measurements were obtained on a StepOne Plus instrument (Applied Biosystems), and data were transferred to an Excel sheet for statistical analyses.

### Statistical analyses

Adapter-trimmed and aligned FASTQ files obtained from the Illumina HiSeq2500 sequencer, by sequencing of our small-RNA libraries (*n* = 18 samples per library), were processed using the RNAworld server from the Tuschl Laboratory at the Rockefeller University. Dedicated Bioconductor packages in the R platform were used to perform statistical analyses of miRNA read counts. The evaluation of differential expression for miRNA data obtained by small-RNA sequencing of our libraries was determined using DESeq2 and edgeR, which included a batch variable to decrease batch bias. For targeted RT-qPCR validations and analyses, the cycle threshold (Ct) value of the endogenous control, RNU6B, was subtracted from the sample miRNA Ct, and changes were calculated using the ∆∆Ct comparative method. RT-qPCR differential expression was assessed via t tests, or Mann–Whitney U tests, of the ∆∆Ct values [48, 50]. Data are expressed as mean ± standard error of the mean (SEM) where a *p*-value ≤ 0.05 was considered statistically significant. The “miRNA score”, based on RT-qPCR validation (∆∆Ct values) of 5 out of the 15 miRNAs and including miR-135a (pval = 0.05), miR-190b (pval = 0.043), miR-205 (pval = 0.00056), miR-30c (pval = 0.011), and miR-744 (pval = 0.038), was established to maximize the discrimination ability of each miRNA and used as a DCIS sample classifier. For our study, we calculated the “miRNA score” [51] by summing the standardized negative levels or z-values of our five significantly downregulated miRNAs for each individual sample (*n* = 30). Correlations between the miRNA score (or its individual miRNA components) and the Oncotype DX® DCIS risk score were performed using Pearson correlations, and miRNA score versus age was analyzed using Spearman correlations. MiRNAs with significant (*p* < 0.05) correlation with age were plotted versus age-group (≤ 55, 55–70, > 70 years). Additionally, miRNAs showing significant (adjusted *p*-value < 0.05) association with DCIS Oncotype DX® DCIS score and/or with age were validated by RT-qPCR.

## Results

### DCIS sample selection, Oncotype DX® DCIS risk scores, and nuclear grade distribution

**Fig. 1:**
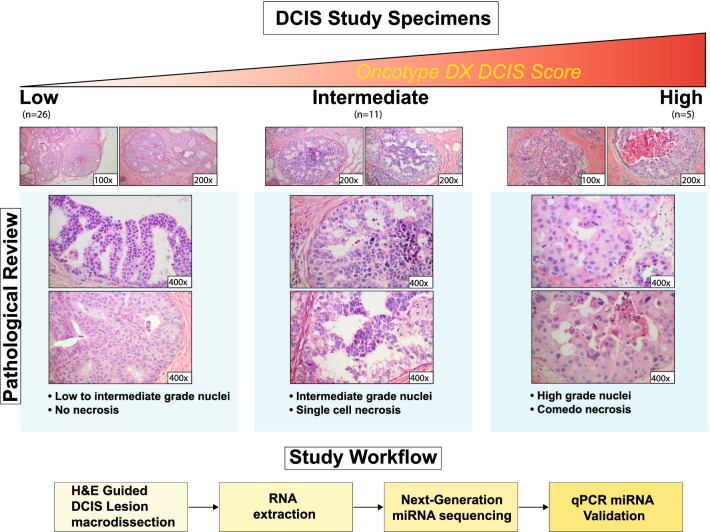
DCIS sample processing and pathology across the Oncotype DX® DCIS score groups. A total of 41 DCIS specimens were selected for the study based on their Oncotype DX® DCIS classification into low (*n* = 26), intermediate (*n* = 10), and high (*n* = 5) risk score groups. Tissue-guided macro-dissection was performed on unstained formalin-fixed paraffin-embedded sections for selection of DCIS lesions prior to RNA extractions. All DCIS lesions evaluated in this study were estrogen receptor (ER) positive

**Fig. 2:**
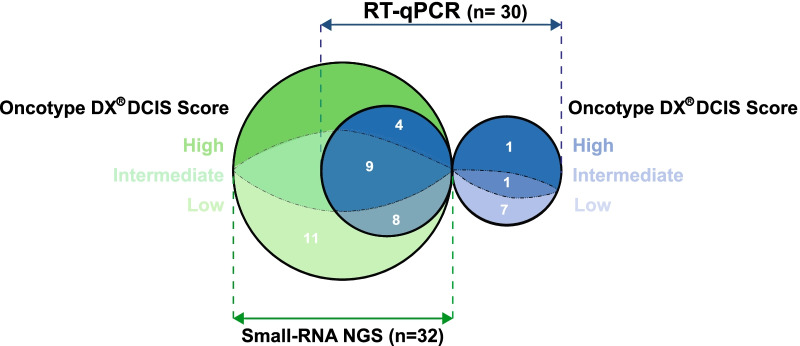
DCIS sample distribution across the three Oncotype DX® DCIS risk groups between NGS and RT-qPCR molecular analyses. The Venn diagram displays the distribution of the individual DCIS samples between next-generation sequencing (NGS) in green and RT-qPCR analyses in blue. The three Oncotype DX® DCIS risk groups, including the low- (light green for NGS and light blue for RT-qPCR), intermediate- (mild green for NGS and mild blue for RT-qPCR), and high-risk (dark green for NGS and dark blue for RT-qPCR) groups separate each circle in three. The number of DCIS RNA samples per analysis (NGS or RT-qPCR) and per Oncotype DX® DCIS risk group are displayed in white in the different circles. Samples utilized for both assays are numbered in areas of the circles that intersect

Following evaluation of the clinical specimens by pathological review (Fig. 1) and Oncotype DX® DCIS scoring, a total of 41 archived FFPE DCIS specimens were selected for this study (26 low, 10 intermediate, and 5 high Oncotype DX® DCIS scores). H&E tissue-guided macro-dissection was performed on the individual lesions in order to enrich our extractions with DCIS lesion RNA (Fig. 1). All DCIS specimens selected for this study were estrogen receptor (ER) positive. The individual Oncotype DX® DCIS scores for the 41 selected samples varied between 0 and 78, as shown in Table 1, where the nuclear grade of the DCIS lesions, patient follow-up data, treatment regimen, and recurrences are also detailed. The nuclear grade distribution of the DCIS samples displayed a correlation with the Oncotype DX® DCIS risk score groups when both intermediate and high-risk score groups were combined (See Additional file 1: Fig. 1). For this study, the mean age of the DCIS patients was 63.5 years old (Oncotype DX® DCIS low (64.8 years old), intermediate (57.9 years old), and high (56.4 years old) risk scores), with an overall median of 66 years (Oncotype DX® DCIS low (67 years old), intermediate (66.5 years old), and low (55 years old) risk scores), based on the ages provided in Table 1. The distribution of the DCIS samples, by Oncotype DX® DCIS risk group (i.e., low, intermediate, and high risk), and between NGS and RT-qPCR experiments is displayed in Fig. 2. For our molecular analyses, we initially performed small-RNA next-generation sequencing (NGS) on 32 (Oncotype DX® DCIS high (*n* = 4), intermediate (*n* = 9), and low (*n* = 19) scores) out of the 41 DCIS samples. Thirty (Oncotype DX® DCIS high (*n* = 5), intermediate (*n* = 10), and low (*n* = 15) scores) out of the 41 study samples (Fig. 2) were used for RT-qPCR validations.

**Table 1:** Clinical information on the DCIS specimens

Oncotype DX DCIS risk score Group	Low							Intermediate							High						
**Patient**	ID	**DX** **Score**	Age	NG	**FU** **Years**	**Treatment**	**Recurrence**	ID	**DX** **Score**	Age	NG	**FU** **Years**	**Treatment**	**Recurrence**	ID	**DX** **Score**	Age	NG	**FU** **Years**	**Treatment**	**Recurrence**
	**DCIS-L1**	11	76	N1	10 ys	RT and AI	NED	**DCIS-I1**	42	78	N2	7 ys	N/A	NED	**DCIS-H1**	56	45	N2	7.5 yr	RT	NED
	**DCIS-L2**	0	72	N1	1 ys	N/A	NED	**DCIS-I2**	47	69	N3	8 ys	RT and TAM	NED	**DCIS-H2**	78	52	N2	8 yr	RT and TAM	NED
	**DCIS-L3**	27	74	N2	5.2 ys	RT and AI	Cntl. DCIS (2y)	**DCIS-I3**	52	66	N2	8 ys	RT and AI	NED	**DCIS-H3**	64	55	N3	6 yr	RT and A	NED
	**DCIS-L4**	0	62	N3	5 ys	no RT, TAM	NED	**DCIS-I4**	48	59	N3	8 ys	RT and AI	NED	**DCIS-H4**	73	72	N3	3 yr	RT and A	NED
	**DCIS-L5**	8	<=30	N2	4.4 ys	None	NED	**DCIS-I5**	39	67	N3	N/A	N/A	N/A	**DCIS-H5**	55	58	N3	6 yr	RT	NED
	**DCIS-L6**	27	65	N3	4 ys	RT and TAM	NED	**DCIS-I6**	40	64	N2	N/A	RT and TAM	N/A	–	–	–	–	–	–	–
	**DCIS-L7**	33	75	N2	2 ys	None	Ipsi. DCIS (2y)	**DCIS-I757**	39	34	N3	MX	MX	MX	–	–	–	–	–	–	–
	**DCIS-L8**	28	62	N2	8 ys	RT and AI	NED	**DCIS-I8**	47	57	N2	MX	MX	MX	–	–	–	–	–	–	–
	**DCIS-L9**	23	68	N1	8 ys	TAM, no RT	NED	**DCIS-I9**	40	76	N3	2 ys	RT	Ipsi. IBC (2y)	–	–	–	–	–	–	–
	**DCIS-L10**	12	66	N2	N/A	N/A	N/A	**DCIS-I10**	52	67	N3	4 ys	AI	NED	–	–	–	–	–	–	–
	**DCIS-L11**	5	70	N1	N/A	N/A	N/A	–	–	–	–	–	–	–	–	–	–	–	–	–	–
	**DCIS-L12**	1	72	N2	8 ys	No RT	NED	–	–	–	–	–	–	–	–	–	–	–	–	–	–
	**DCIS-L13**	5	76	N2	4 ys	No RT	NED	–	–	–	–	–	–	–	–	–	–	–	–	–	–
	**DCIS-L14**	13	68	N3	7 ys	AI, no RT	NED	–	–	–	–	–	–	–	–	–	–	–	–	–	–
	**DCIS-L15**	20	66	N2	6 ys	AI, no RT	NED	–	–	–	–	–	–	–	–	–	–	–	–	–	–
	**DCIS-L16**	22	42	N2	N/A	N/A	N/A	–	–	–	–	–	–	–	–	–	–	–	–	–	–
	**DCIS-L17**	37	61	N2	6 ys	RT and AI	NED	–	–	–	–	–	–	–	–	–	–	–	–	–	–
	**DCIS-L18**	15	40	N2	5 ys	N/A	NED	–	–	–	–	–	–	–	–	–	–	–	–	–	–
	**DCIS-L19**	15	65	N2	4.5 ys	AI, no RT	NED	–	–	–	–	–	–	–	–	–	–	–	–	–	–
	**DCIS-L20**	0	72	–	4 ys	AI, no RT	NED	–	–	–	–	–	–	–	–	–	–	–	–	–	–
	**DCIS-L21**	0	62	N1	N/A	N/A	N/A	–	–	–	–	–	–	–	–	–	–	–	–	–	–
	**DCIS-L22**	24	>=80	N3	4.5 ys	RT and TAM	NED	–	–	–	–	–	–	–	–	–	–	–	–	–	–
	**DCIS-L23**	36	79	N3	4 ys	None	NED	–	–	–	–	–	–	–	–	–	–	–	–	–	–
	**DCIS-L24**	0	44	N1	10 ys	TAM, no RT	NED	–	–	–	–	–	–	–	–	–	–	–	–	–	–
	**DCIS-L25**	6	73	N2	N/A	N/A	N/A	–	–	–	–	–	–	–	–	–	–	–	–	–	–
	**DCIS-L26**	28	64	N2	5 ys	AI, no RT	NED	–	–	–	–	–	–	–	–	–	–	–	–	–	–

Forty-one DCIS specimens were selected for this study and were separated in three groups including low (*n* = 26), intermediate (*n* = 10), and high (*n* = 5) Oncotype DX® DCIS risk scores. Patient identification (ID), age, Oncotype DX® DCIS score and nuclear grade of the DCIS lesions, treatment and recurrence data are provided in the table

*DX Score* Oncotype DX **®** DCIS score

*N* Nuclear grade (N1—low, N2—intermediate, N3—high)

*FU Years* Follow-up (FU) in years (ys), mastectomy (MX)

*Treatment* No Therapy (none), Mastectomy (MX), Radiotherapy (RT), Aromatase Inhibitors (AI), Tamoxifen (TAM)

*Recurrence* No evidence of disease (NED), ipsilateral DCIS (Ipsi. DCIS), ipsilateral invasive breast cancer (Ipsi. IBC), contralateral DCIS (Cntl. DCIS)

### miRNA next-generation sequencing (NGS) results correlate with Oncotype DX® DCIS score subgroup distribution

**Fig. 3:**
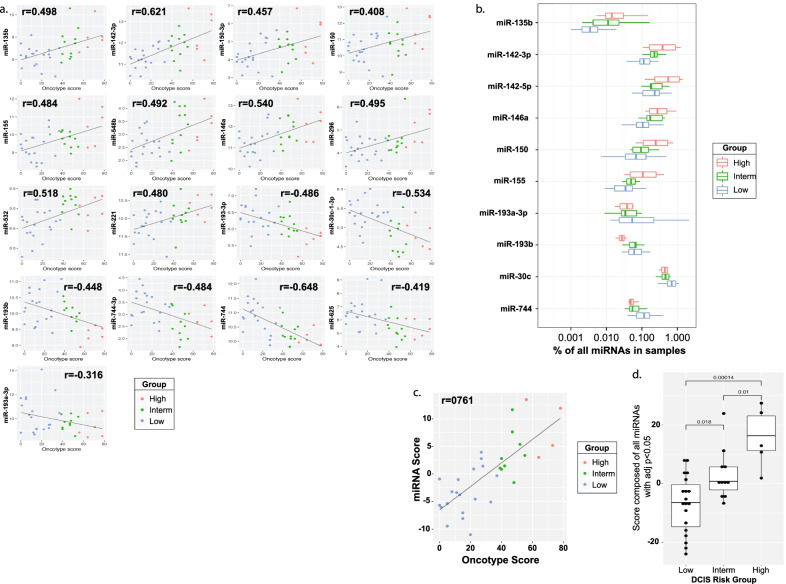
Correlation of top differentially expressed miRNAs with the three Oncotype DX® DCIS risk groups. Four small-RNA cDNA libraries were prepared using RNA samples collected from 32 individual FFPE DCIS specimens, with each library having an even representation of low-, intermediate-, and high-risk score Oncotype DX® DCIS RNA samples. **a** Individual box plot analyses of 17 differentially expressed miRNAs displaying a correlation with the different Oncotype DX® DCIS risk groups. **b** Box plot representation of the miRNA reads for the top 10 differentially expressed miRNAs and with distribution in each of the three Oncotype DX® DCIS risk groups. **c** Comparison of the miRNA composite risk score (obtained from the top 17 differentially expressed miRNAs) to the Oncotype DX® DCIS risk scores of the 32 DCIS samples evaluated by next-generation sequencing (NGS). **d** Box plot representation of the miRNA composite risk score for each individual Oncotype DX® DCIS risk groups, displaying significant miRNA expression differences between the low and intermediate (*p* < 0.018), intermediate and high (*p* < 0.01), and low and high (*p* < 0.00014) risk groups

Small-RNA cDNA libraries were prepared using 32 individual RNA samples from FFPE DCIS specimens (samples were arranged in 4 distinct libraries) with each library having an even representation of low-, intermediate- and high-risk score Oncotype DX^®^ DCIS RNA samples, to minimize batch effects. Our small-RNA expression analyses identified 17 differentially expressed miRNAs displaying a correlation with the three different Oncotype DX^®^ DCIS scores (Fig. 3a). Of which, 10 miRNAs, namely miR-135b (*r* = 0.498), miR-142-3p (*r* = 0.621), miR-150-3p (*r* = 0.457), miR-150 (*r* = 0.408), miR-155 (*r* = 0.484), miR-548b (*r* = 0.492), miR-146a (*r* = 0.540), miR-296-5p (*r* = 0.495), miR-532-5p (*r* = 0.518) and miR-221 (*r* = 0.480), displayed a positive correlation, and 7 miRNAs, namely miR-193b-5p (*r* = − 0.486), miR-30c-1-3p (*r* = − 0.534), miR-193b (*r* = − 0.448), miR-744-3p (*r* = − 0.484), miR-744 (*r* = − 0.648), miR-625-5p (*r* = − 0.419) and miR-193a-5p (*r* = − 0.316), which displayed a negative correlation with the Oncotype DX^®^ DCIS score groups. As presented in Fig. 3b, the top 9 most differentially expressed miRNAs represented between 0.1 and 1% of all miRNAs detected by small-RNA sequencing, with one miRNA (hsa-miR-135b) in low abundance but reproducibly detectable across all three Oncotype DX^®^ DCIS groups. In order to maximize miRNA discrimination ability, we established a composite score using the top 17 differentially expressed miRNAs and plotted our “miRNA score” versus the “Oncotype score” on the 32 sequenced DCIS samples. A positive correlation (*r* = 0.761; Pearson correlation analysis) was observed between the miRNA score and the Oncotype score (Fig. 3c) with a clear distinction being observed between low, intermediate (*p* = 0.018 compared to low), and high (*p* = 0.00014 compared to low and *p* = 0.01 compared to intermediate) Oncotype DX^®^ DCIS risk score groups (Fig. 3d).

### miRNA expression deregulation in DCIS lesions correlates with patient age:

**Fig. 4:**
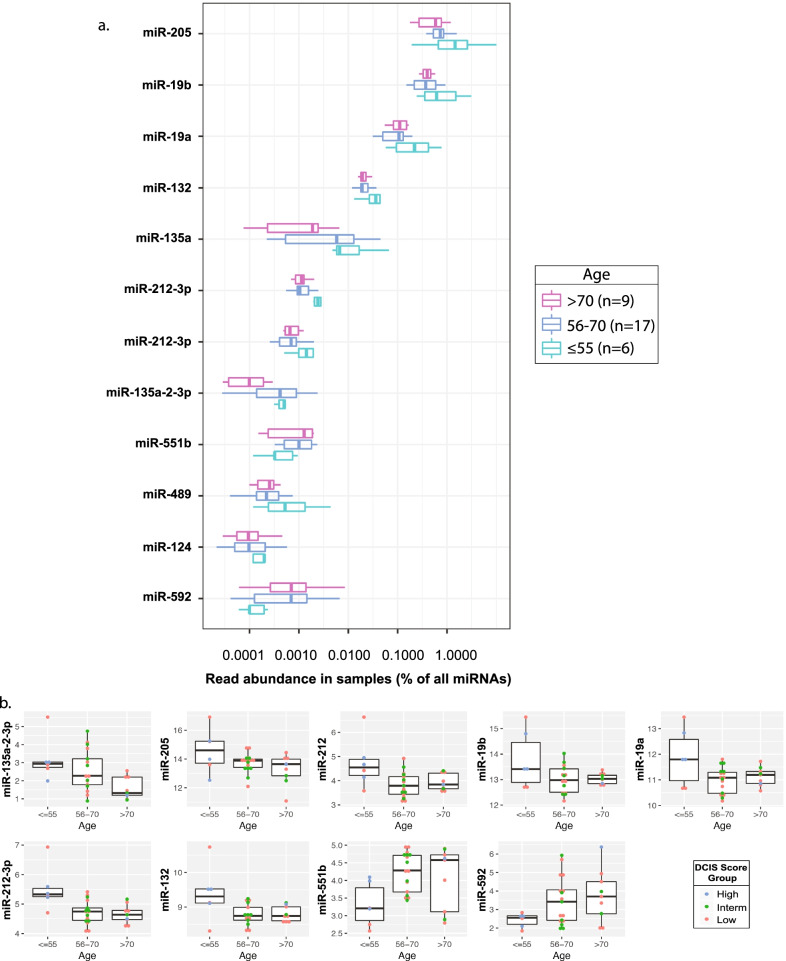
Correlation of top differentially expressed miRNAs with patient age at the time of Oncotype DX® DCIS testing. **a** Box plot distribution of the top 12 differentially expressed miRNAs between three age-groups (groups#1 ≤ 55 (*n* = 6), group#2 56–70 (*n* = 17), and group#3 > 70 (*n* = 9) years old) with percentage of reads per miRNA. **b** Box plot representations of the top 9 differentially expressed miRNAs in the DCIS lesions from patients separated in the three different age-groups, with decreasing expression trends for 7 miRNAs (hsa-miR-135a-2-3p, hsa-miR-205, hsa-miR-212-5p, hsa-miR-19b, hsa-miR-19a, hsa-miR-212-3p, and hsa-miR-132), and an increasing expression trend for hsa-miR-551b and hsa-miR-592

Using our small-RNA NGS data, we next sought to evaluate the correlation between miRNA expression changes and the age of patients at the time of Oncotype DX^®^ DCIS assay testing. When patients were subgrouped into the three different age categories, namely ≤55 (*n*=6), 56–70 (*n*=17) and >70 (*n*=9) years old (see ages in Table 1), we observed 12 differentially expressed miRNAs, which were reproducibly expressed and detectable across all three age-groups with abundance varying between 0.0001 and 1% of all miRNA reads (Fig. 4a). We performed Spearman correlation analyses and demonstrated that with increasing age a significant decrease in miRNA expression was observable for seven miRNAs including miR-135a-2-3p (adj pval=0.0269), miR-205 (adj pval=0.0020), miR-212-5p (adj pval=0.0016), miR-19b (adj pval=0.0038), miR-19a (adj pval=0.0016), miR-212-3p (adj pval=0.0016) and miR-132 (adj pval=0.0044), whereas an increase in miRNA expression was only observed for two miRNAs, including miR-551b (pval<0.0440) and miR-592 (adj pval=0.0214) (Fig. 4b). The adjusted p values for miRNA expression decrease were overall much lower than those for the miRNAs expression increase with age INCREASE.

### qPCR validation of top differentially expressed miRNAs between Oncotype DX® DCIS risk groups

**Fig. 5:**
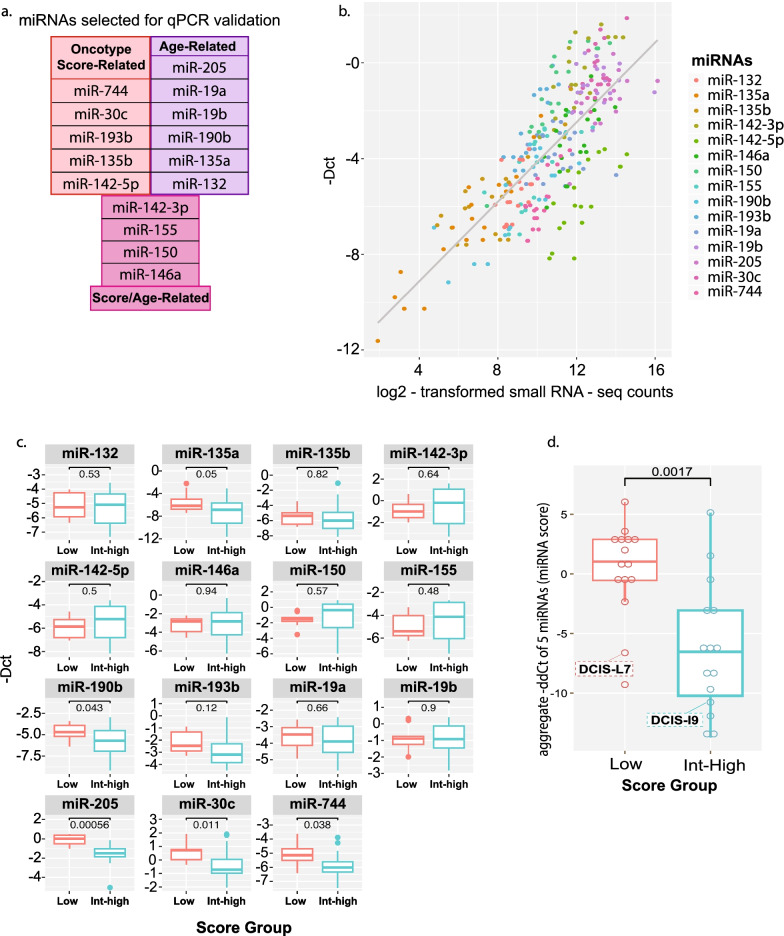
RT-qPCR validation of the top 15 differentially expressed miRNAs between Oncotype DX® DCIS risk groups based on expression levels and age of patients. **a** Five Oncotype DX**®** DCIS risk score-related miRNAs, six age-related miRNAs, and four miRNAs based on a combination of Oncotype DX**®** DCIS risk score-related and patient age-related, identified by NGS analyses, were selected for RT-qPCR validations. **b** A positive correlation was observed for log2-transformed small-RNA sequencing read counts and RT-qPCR Ct values obtained for the top 15 differentially expressed miRNAs evaluated using RNA samples from DCIS lesions classified with Oncotype DX DCIS assay (see Fig. 2, RT-qPCR samples (*n* = 30). **c** Individual box plot evaluations of the 15 selected miRNAs measured by RT-qPCR in 30 samples (See Fig. 2), between low and high/intermediate Oncotype DX**®** DCIS risk score groups for identification of the top 5 differentially expressed miRNAs between the two groups (miR-135a, miR-190b, miR-205, miR-30c and miR-744). **d** Composite RT-qPCR miRNA score based on RT-qPCR expression of the 5 differentially expressed miRNAs. A highly significant expression difference (*p* < 0.0017) was obtained between the two Oncotype DX**®** DCIS risk groups (low and intermediate/high groups). The red dashed rectangle, in the low composite miRNA group (left), identifies DCIS specimen (i.e., Oncotype DX**®** DCIS low-risk score of 33 in Table 1) from patient DCIS-L7 who experienced an ipsilateral DCIS recurrence after 2 years of the initial DCIS diagnosis. The blue dashed rectangle, in the Int-High miRNA group (right), identifies DCIS specimen (i.e., Oncotype DX® DCIS intermediate risk score of 40 in Table 1), from the patient DCIS-I9 who experienced an ipsilateral invasive breast cancer recurrence after 2 years of the initial DCIS diagnosis

Considering that our NGS observations indicated that specific miRNA expression changes (i.e., up- or downregulation) correlated with an increase in the Oncotype DX® DCIS risk scores, patient age, or both, we sought to confirm our findings by using gold standard miRNA RT-qPCR assays, and thus selected the top 15 most significantly (*p* < 0.05) differentially expressed miRNAs for these validations. We selected 5 Oncotype DX® DCIS score-related miRNAs, namely miR-744, miR-30c, miR-193b, miR-135b, miR-142-5p, 6 age-related miRNAs; miR-205, miR-19a, miR-19b, miR-190b, miR-135a and miR-132, and 4 miRNAs; miR-155, miR-150, miR-146a, miR-142-3p, which displayed associations with both the Oncotype DX® DCIS score and patient age (Fig. 5a). A strong positive correlation was observed for log2-transformed small-RNA NGS counts and RT-qPCR Ct values for the top 15 differentially expressed miRNA (Fig. 5b), which confirmed the specificity of our NGS measurements. Our RT-qPCR data also revealed that five miRNAs, namely miR-135a (pval = 0.05), miR-190b (pval = 0.043), miR-205 (pval = 0.00056), miR-30c (pval = 0.011), and miR-744 (pval = 0.038) displayed a significantly decreased expression when compared to increased Oncotype DX® DCIS risk scores (Fig. 5c).

### qPCR miRNA composite score separates Oncotype DX® DCIS risk scores in two groups

The assessment of our miRNA composite score that included the 5 topmost differentially expressed and RT-qPCR validated miRNAs (i.e., see Statistical section for calculation details on composite miRNA score) identified a trend of overall significant decrease in miRNA expression between the low and the intermediate/high Oncotype DX® DCIS risk score groups (Fig. 5d, *p*  = 0.0017), correlating with increase in the Oncotype DX® DCIS risk scores. Since we designed our miRNA composite risk score to correlate with the Oncotype DX® DCIS risk score, we did not observe a correlation with the nuclear grade of the DCIS specimens (See Additional file 1: Fig. 1c. *r *= 0.168) for which the Oncotype DX® DCIS assay displayed a low correlation (See Additional file 1: Fig. 1b  * r* = 0.509). When evaluating our patient follow-up data, we determined that two patients in our study experienced an ipsilateral recurrence 2 years after their initial DCIS diagnosis (i.e., one DCIS and one invasive breast cancer recurrence; See Table 1). The DCIS lesion of the patient (i.e., DCIS-L7 in Table 1.) who experienced a subsequent ipsilateral DCIS recurrence was initially provided a low Oncotype DX® DCIS risk score of 33, but classified as the 2nd most extreme miRNA composite score in our miRNA low score group (Fig. 5d, red dashed rectangle). The position of this composite miRNA score was equivalent to the median position in the Int-High miRNA composite score. The DCIS lesion of the patient (i.e., DCIS-I9 in Table 1.) who experienced a subsequent ipsilateral invasive breast cancer recurrence (i.e., 2 years after initial DCIS diagnosis) was initially provided an intermediate Oncotype DX® DCIS risk score of 44, but classified as the 4^th^ most extreme miRNA composite score in our high-Int miRNA score group (Fig. 5d, blue dashed rectangle). However, our data suggest that the analysis of our small panel of 5 miRNAs may provide additional molecular information for the clinical evaluation of DCIS lesions.

## Discussion

Advancements in modern high-quality screening and diagnostic mammography are widely acknowledged to have resulted in a significant increase in the detection and diagnosis of ductal carcinoma *in situ* (DCIS) [52]. The likelihood of DCIS recurrence or the progression to invasive breast cancer (IBC), however, is poorly understood, and for women diagnosed with DCIS, it remains difficult to predict who will go on to recur and/or to develop IBC [53–55]. As such, the 12-gene DCIS Oncotype DX^®^ assay was developed and has been shown to allow for a more comprehensive molecular evaluation of DCIS lesions. This score-based assay, which stratifies patients into either low, intermediate, or high-risk groups, has been shown to successfully evaluate the risk of local recurrence and as such may help guide treatment strategies [56]. Increasingly, more studies have shown that there is a direct relationship between the deregulation of miRNAs and the onset/progression of a variety of cancers [57–59]. In this study we sought to determine the utility of correlating miRNA expression data from archived formalin-fixed paraffin-embedded (FFPE) specimens obtained from women diagnosed with DCIS, whose lesions were evaluated with the Oncotype DX^®^ DCIS assay and with known risk scores. For this retrospective study, we used methodologies we developed to analyze small RNAs, which we demonstrated to be applicable for the analysis of archived FFPE specimens up to 35 years old [48].

Multiple studies have begun to evaluate miRNA expression changes in tumors as diagnostic and prognostic tools [60–64]. Assessment of miRNA expression patterns has shown that they can act as oncogenes or tumor suppressors and display up- or downregulated expression patterns in many human tumors [65]. It has been documented, for example, that DCIS tissue lesions exhibits a general upregulation in miR-21 expression when compared to normal breast tissue [44, 66, 67]. It has been shown that the upregulation of miR-654 in DCIS lesions is associated with poor patient prognosis [68]. Conversely, the expression of miR-125b has been shown to be downregulated in DCIS lesions [69]. In normal breast tissue, the expression of miR-124, miR-145, and miR-205 appears enriched exclusively in the basal compartment of ducts, whereas in matching breast tumors their expression appears to be reduced [70]. When comparing normal breast tissue to atypical ductal hyperplasia (ADH), DCIS lesions, and invasive ductal carcinoma (IDC), Chen et al identified that miR-21, miR-200b/c, miR-183, and miR-141 displayed an increase expression associated with histological progression toward invasive lesions [66]. Altogether, these studies have highlighted the strong potential for using miRNA expression quantification as a means for molecular evaluation of DCIS lesions.

In the current study, we utilized risk scores obtained with the Oncotype DX^®^ DCIS assay as an initial classifier to perform miRNA next-generation sequencing (NGS) of classified DCIS lesions (i.e., high-, intermediate-, and low-risk scores). Our NGS analyses revealed a linear relationship between increased Oncotype DX^®^ DCIS risk scores and the deregulated expression of 15 miRNAs (either up- or downregulation). To further evaluate the robustness of our NGS findings, we quantified the expression of these 15 miRNAs by qRT-PCR using total RNA purified from archived DCIS lesions, classified with the Oncotype DX^®^ DCIS assay. These analyses revealed a strong correlation between expression downregulation of miR-30c, miR-135a, miR-190b, miR-205, and miR-744 and of the risk scores obtained from the Oncotype DX^®^ DCIS assay. These analyses revealed that the highest miRNA expression differences could be observed by combining the intermediate and high-risk groups, in comparison to the low-risk group. Our miRNA composite risk score based on the validated expression of these 5 miRNAs further highlighted the molecular difference between low- and intermediate-/high-risk groups (*p*<0.0021). These findings are in agreement with the original study used to develop the Oncotype DX^®^ DCIS Assay (ECOG 5194) where the risk of local recurrence between the intermediate and low risk was higher than between high-risk and low-risk groups [27, 36]. More precisely, the analyses of Solin et al. revealed that the 10-year risk of developing IBC was 10.6% for the low-risk group, 26.7% for the intermediate risk group, and 25.9% for the high-risk score group, with the highest recurrence rate for the intermediate group [27]. Incidentally, although our sample size was small (i.e., 41 patients) and our follow-up data (i.e., 32 out of 41 patients) was generally limited to 4–8 years, we identified two patients diagnosed with DCIS who experienced ipsilateral recurrences (i.e., one patient with a DCIS recurrence and one patient with an invasive breast cancer recurrence). The patient who experienced a DCIS recurrence was initially provided a low Oncotype DX^®^ DCIS risk score, but classified with the 2nd most extreme decreased miRNA expression in our low score group. Additionally, the DCIS patient who experienced an invasive breast cancer recurrence and whose DCIS lesion was initially provided an intermediate Oncotype DX^®^ DCIS risk score obtained the 4th most extreme miRNA expression decrease in our intermediate-/high-risk score. Although our sample size was small and only identified two ipsilateral recurrences, our data suggest that the miRNA composite score may have prognostic utility beyond the Oncotype DX^®^ DCIS assay score, a hypothesis that will need to be evaluated in follow-up studies.

As detailed in our study, we identified and validated a subset of five miRNAs for which progressive expression downregulation was significantly associated with increased Oncotype DX^®^ DCIS risk scores, which could suggest that some of these miRNAs may be directly or indirectly involved in the expression regulation of some of the genes included in the Oncotype DX^®^ DCIS assay, but that will require further experimental confirmation. Interestingly, one of these miRNAs, miR-190b (*p*=0.043), has been shown to be the most upregulated miRNA in estrogen receptor-positive cancer [71]. Hsa-miR-30c, which was the second most downregulated miRNA in our study (*p*=0.011), has been reported to be an independent predictor for benefit of endocrine therapy in patients with ER-positive breast cancers. Considering that all DCIS lesions evaluated in our study were ER positive, expression downregulation of hsa-miR-30c may potentially have both prognostic and predictive value, but will require long-term follow-up [72]. In a recent study of ER-positive invasive breast tumors, we also identified downregulated expression of hsa-miR-30c in patient tumors correlated with subsequent metastasis, suggesting that this miRNA could provide information on the potential of DCIS lesions for progression to invasive disease [73]. The expression of miR-744 is known to be downregulated in several cancers including breast tumors [74]. *In vitro* studies evaluating overexpression of hsa-miR-744 in breast cancer cell lines and in particular ER-positive MCF-7 breast cancer cells lead to a significant inhibition of cellular proliferation. For DCIS lesions the decrease in hsa-miR-744 expression may provide a significant benefit for the cellular proliferation of DCIS cells. Interestingly, Chen et al. determined that increased expression of hsa-miR-744 in FFPE specimens from breast cancer patients was associated with chemo-resistance [75]. Finally, multiple molecular expression studies have shown a direct relationship between hsa-miR-135a expression and various human malignancies, including breast cancer [65, 72]. While some contrasting results have been reported, whereby hsa-miR-135a is either up or downregulated in differing cancers, hsa-miR-135a appears to function as a tumor suppressor in breast cancer and seems to be downregulated in human ER+ and triple negative (TN) breast cancers [65, 76, 77].

It is noteworthy that 3 of the 5 miRNAs in our composite risk score, specifically miR-205, miR-190b, and miR-135a, were derived from our analysis of the correlation between their expression in the DCIS lesions and the patient’s age. The original Oncotype DX^®^ DCIS assay, which was established after analysis of local recurrences in 327 patients with DCIS in the ECOG 5194 study, provided recurrence risk estimates solely based on the DCIS Score [27]. However, a more recent multivariable analysis (2018) performed on a larger cohort (Ontario cohort), which included 571 women diagnosed with DCIS found that after adjustment for clinicopathologic variables, particularly patient age, this had a larger effect on the evaluation of local recurrence risk than the DCIS score alone [36, 78, 79]. Our findings would further confirm that patients’ age and in turn miRNA expression changes associated with age in those patients provide additional targets for the evaluation of the patient’s risk. Currently, the local recurrence risk algorithm provided by the DCIS score incorporates patient age and varies more due to patient age than the actual score, with patients under 50 reported as having a higher local recurrence risk at any score. In our study, all cases had a DCIS score reported prior to incorporation of age in recurrence risk estimates; however, our analyses would suggest that these age-related miRNA expression changes might help refine the Oncotype DX^®^ DCIS local recurrence risk estimates.

The most significant result drawn from this study was the highly significant decrease in expression of hsa-miR-205 (adj *p*<0.00056 by RT-qPCR validation) in DCIS lesions with intermediate/high Oncotype DX^®^ DCIS scores. Hsa-miR-205 has already been demonstrated to confer invasive potential to DCIS cells in a breast cancer cell progression model described by Chen et al. [78]. Hsa-miR-205 has recently been described as an oncosuppressive miRNA, because its expression is directly regulated by the tumor suppressor p53 [78–80]. Mutations in p53 have been shown to inhibit the expression of hsa-miR-205, leading to enhanced cellular proliferation of breast cancer cells [80]. Interestingly, hsa-miR-205 has also been found to target the human epidermal growth factor receptor, HER3, in human ER+ breast cancer cells [80]. Furthermore, others have found hsa-miR-205 binding sites in the noncoding region of the Zinc Finger E-Box Binding Homeobox 1 (Zeb1) and Smad interacting protein 1 (Sip1) mRNA sequences, two key proteins, which have been implicated in epithelial–mesenchymal transition (EMT) and suggesting a potential role for DCIS to invasive breast cancer cell transition/progression [81]. In vitro studies in MDCK–Pez cells (a canine epithelial cell line that has undergone EMT via the overexpression of protein tyrosine phosphatase Pez) further highlighted that addition of exogenous precursor hsa-miR-205 results in a phenotypic change from mesenchymal-like back to epithelial-like [81]. Altogether, decreased expression of hsa-miR-205 is in agreement with the literature, whereby hsa-miR-205 is consistently downregulated in breast cancers [78–81], suggesting that early molecular changes such as decreased expression of hsa-miR-205, as early as in DCIS lesions, may increase the likeliness of breast cancer progression. However, larger-scale and more focused studies will be required to evaluate the prognostic potential of hsa-miR-205 and other miRNAs.

## Conclusion

Taken together, our data suggest that miRNA expression analyses of DCIS lesions, when compared to the Oncotype DX^®^ DCIS Assay, have potential to provide important prognostic information on the risk of local recurrence. Considering that some of the deregulated miRNAs, identified in our study, have been showing promise as prognostic and predictive biomarkers for breast cancer, further investigations are required to determine their potential for the accurate evaluation of the recurrence risk and particularly as molecular guides for clinical management of DCIS. Finally, our robust data demonstrate that miRNA high-throughput analyses can be performed in a retrospective context but that larger-scale studies are required for biomarker discovery.

## Supplementary Information

**Additional file 1**.** Fig. 1**: Correlation between nuclear grade of the DCIS specimens and the Oncotype DX^®^ DCIS risk score or the miRNA composite score. **a**. Result tables displaying distribution of the nuclear grades of 40 out of the 41 DCIS samples (One Low Oncotype DX^®^ DCIS risk score sample was missing nuclear grade) by Oncotype DX^®^ DCIS risk scores. The left table displays the distribution of the nuclear grades of the DCIS samples between the three Oncotype DX^®^ DCIS risk score groups (i.e., low-, intermediate-, and high-risk scores), providing a nonsignificant p value of 0.07906. The right table displays distribution of the nuclear grade of the DCIS samples between the low- and the intermediate-/high-risk score groups, which provides a significant p value of 0.01525. **b**. correlation plot between the DCIS sample’s Oncotype DX^®^ DCIS risk scores and the three nuclear grades (Pearson correlation coefficient (Rho) r = 0.506). **c**. Correlation plot between the DCIS sample’s miRNA composite risk scores and the three nuclear grades (Pearson correlation coefficient (Rho) r = 0.168).

## Data Availability

In order to protect confidential patient information, datasets generated and analyzed in this study will not be made publicly available.

## References

[1] Vaidya Y, Vaidya P, Vaidya T (2015). Ductal carcinoma in situ of the breast. Indian J Surg.

[2] Allred DC (2010). Ductal carcinoma in situ: terminology, classification, and natural history. J Natl Cancer Inst Monogr.

[3] Ward WH, DeMora L, Handorf E (2020). Preoperative delays in the treatment of DCIS and the associated incidence of invasive breast cancer. Ann Surg Oncol.

[4] Siegel RL, Miller KD, Jemal A (2018). Cancer statistics, 2018. CA Cancer J Clin.

[5] Kong J, Liu X, Zhang X, Zou Y (2020). The predictive value of calcification for the grading of ductal carcinoma in situ in Chinese patients. Medicine (Baltimore).

[6] Sundara Rajan S, Verma R, Shaaban AM, Sharma N, Dall B, Lansdown M (2013). Palpable ductal carcinoma in situ: analysis of radiological and histological features of a large series with 5-year follow-up. Clin Breast Cancer.

[7] Gooch JC, Chun J, Kaplowitz E, Kurz E, Guth A, Lee J, Schnabel F (2019). Breast density in a contemporary cohort of women with ductal carcinoma in situ (DCIS). Ann Surg Oncol.

[8] Erbas B, Provenzano E, Armes J, Gertig D (2006). The natural history of ductal carcinoma in situ of the breast: a review. Breast Cancer Res Treat.

[9] Wang LC, Sullivan M, Du H, Feldman MI, Mendelson EB (2013). US appearance of ductal carcinoma in situ. Radiographics.

[10] Parikh U, Chhor CM, Mercado CL (2018). Ductal carcinoma in situ: the whole truth. AJR Am J Roentgenol.

[11] Li CI, Daling JR, Malone KE (2005). Age-specific incidence rates of in situ breast carcinomas by histologic type, 1980 to 2001. Cancer Epidemiol biomark prev publ Am Assoc Cancer Res Cosponsored Am Soc Prev Oncol.

[12] Virnig BA, Tuttle TM, Shamliyan T, Kane RL (2010). Ductal carcinoma in situ of the breast: a systematic review of incidence, treatment, and outcomes. J Natl Cancer Inst.

[13] Page DL, Dupont WD, Rogers LW, Jensen RA, Schuyler PA (1995). Continued local recurrence of carcinoma 15–25 years after a diagnosis of low grade ductal carcinoma in situ of the breast treated only by biopsy. Cancer.

[14] Collins LC, Tamimi RM, Baer HJ, Connolly JL, Colditz GA, Schnitt SJ (2005). Outcome of patients with ductal carcinoma in situ untreated after diagnostic biopsy: results from the Nurses' Health Study. Cancer.

[15] Williams KE, Barnes NLP, Cramer A, Johnson R, Cheema K, Morris J, Howe M, Bundred NJ (2015). Molecular phenotypes of DCIS predict overall and invasive recurrence. Ann Oncol.

[16] Giannakeas V, Sopik V, Narod SA (2018). A comparison of two models for breast cancer mortality for women with ductal carcinoma in situ: an SEER-based analysis. Breast Cancer Res Treat.

[17] Giannakeas V, Sopik V, Narod SA (2020). Association of a diagnosis of ductal carcinoma in situ with death from breast cancer. JAMA Netw Open.

[18] Tavassoli FA (2005). Breast pathology: rationale for adopting the ductal intraepithelial neoplasia (DIN) classification. Nat Clin Pract Oncol.

[19] Pinder SE (2010). Ductal carcinoma in situ (DCIS): pathological features, differential diagnosis, prognostic factors and specimen evaluation. Mod Pathol.

[20] Zhang X, Dai H, Liu B, Song F, Chen K (2016). Predictors for local invasive recurrence of ductal carcinoma in situ of the breast: a meta-analysis. Eur J Cancer Prev.

[21] Sanders ME, Schuyler PA, Dupont WD, Page DL (2005). The natural history of low-grade ductal carcinoma in situ of the breast in women treated by biopsy only revealed over 30 years of long-term follow-up. Cancer.

[22] Yeong J, Thike AA, Tan PH, Iqbal J (2017). Identifying progression predictors of breast ductal carcinoma in situ. J Clin Pathol.

[23] Correa C, McGale P (2010). Overview of the randomized trials of radiotherapy in ductal carcinoma in situ of the breast. J Natl Cancer Inst Monogr.

[24] Giannakeas V, Sopik V, Narod SA (2018). Association of radiotherapy with survival in women treated for ductal carcinoma in situ with lumpectomy or mastectomy. JAMA Netw Open.

[25] Rudloff U, Jacks LM, Goldberg JI (2010). Nomogram for predicting the risk of local recurrence after breast-conserving surgery for ductal carcinoma in situ. J Clin Oncol.

[26] Kerlikowske K, Molinaro AM, Gauthier ML, Berman HK, Waldman F, Bennington J, Sanchez H, Jimenez C, Stewart K, Chew K, Ljung BM, Tlsty TD (2010). Biomarker expression and risk of subsequent tumors after initial ductal carcinoma in situ diagnosis. J Natl Cancer Inst.

[27] Solin LJ (2013). A Multigene expression assay to predict local recurrence risk for ductal carcinoma in situ of the breast. J Natl Cancer Inst.

[28] Whitney J, Corredor G, Janowczyk A, Ganesan S, Doyle S, Tomaszewski J, Feldman M, Gilmore H, Madabhushi A (2018). Quantitative nuclear histomorphometry predicts oncotype DX risk categories for early stage ER+ breast cancer. BMC Cancer.

[29] Durrani S, Al-Mushawa F, Heena H, Wani T, Al-Qahtani A (2021). Relationship of oncotype Dx score with tumor grade, size, nodal status, proliferative marker Ki67 and nottingham prognostic index in early breast cancer tumors in Saudi population. Ann Diagn Pathol.

[30] Huang Z, Qin Q, Xia L, Lian B, Tan Q, Yu Y, Mo Q (2021). significance of oncotype DX 21-gene test and expression of long non-coding RNA MALAT1 in early and estrogen receptor-positive breast cancer patients. Cancer Manag Res.

[31] Baehner FL (2016). The analytical validation of the oncotype DX Recurrence Score assay. Ecancermedicalscience.

[32] Pennock ND, Jindal S, Horton W, Sun D, Narasimhan J, Carbone L, Fei SS, Searles R, Harrington CA, Burchard J, Weinmann S, Schedin P, Xia Z (2019). RNA-seq from archival FFPE breast cancer samples: molecular pathway fidelity and novel discovery. BMC Med Genomics.

[33] Cohen PA, Loudig O, Liu C, Albanese J, Fineberg S (2019). The ZNF217 biomarker predicts low- and high-risk oncotype DX® recurrence score in ER-positive invasive breast cancers. Front Pharmacol.

[34] Nofech-Mozes S, Hanna W, Rakovitch E (2019). Molecular evaluation of breast ductal carcinoma in situ with oncotype DX DCIS. Am J Pathol.

[35] Paszat L, Sutradhar R, Zhou L, Nofech-Mozes S, Rakovitch E (2018). Including the ductal carcinoma in situ (DCIS) Score in the development of a multivariable prediction model for recurrence after excision of DCIS. Clin Breast Cancer.

[36] Rakovitch E, Gray R, Baehner FL (2018). Refined estimates of local recurrence risks by DCIS score adjusting for clinicopathological features: a combined analysis of ECOG-ACRIN E5194 and Ontario DCIS cohort studies. Breast Cancer Res Treat..

[37] Bartel D (2004). MicroRNAs: genomics, biogenesis, mechanism, and function. Cell Press.

[38] Cannell IG, Kong YW, Bushell M (2008). How do microRNAs regulate gene expression?. Biochem Soc Trans.

[39] Hobert O (2008). Gene regulation by transcription factors and microRNAs. Science.

[40] Gong X, Sun J, Zhao Z (2011). Gene regulation in glioblastoma: a combinatorial analysis of microRNAs and transcription factors. Int J Comput Biol Drug Des.

[41] Selbach M, Schwanhäusser B, Thierfelder N, Fang Z, Khanin R, Rajewsky N (2008). Widespread changes in protein synthesis induced by microRNAs. Nature.

[42] Uhlmann S, Mannsperger H, Zhang JD, Horvat EÁ, Schmidt C, Küblbeck M, Henjes F, Ward A, Tschulena U, Zweig K, Korf U, Wiemann S, Sahin O (2012). Global microRNA level regulation of EGFR-driven cell-cycle protein network in breast cancer. Mol Syst Biol.

[43] York A, Teo T (2021). Tiny MiRNAs play a big role in the treatment of breast cancer metastasis. Cancers.

[44] Hannafon BN, Ding WQ (2018). MiRNAs as biomarkers for predicting the progression of ductal carcinoma in situ. Am J Pathol.

[45] Zhang B, Pan X, Cobb GP, Anderson TA (2007). MicroRNAs as oncogenes and tumor suppressors. Dev Biol.

[46] Loudig O, Milova E, Brandwein-Gensler M, Massimi A, Belbin TJ, Childs G, Singer RH, Rohan T, Prystowsky MB (2007). Molecular restoration of archived transcriptional profiles by complementary-template reverse-transcription (CT-RT). Nucleic Acids Res.

[47] Loudig O, Brandwein-Gensler M, Kim RS, Lin J, Isayeva T, Liu C, Segall JE, Kenny PA, Prystowsky MB (2011). Illumina whole-genome complementary DNA-mediated annealing, selection, extension and ligation platform: assessing its performance in formalin-fixed, paraffin-embedded samples and identifying invasion pattern-related genes in oral squamous cell carcinoma. Hum Pathol.

[48] Loudig Ol (2017). Evaluation and adaptation of a laboratory-based CDNA library preparation protocol for retrospective sequencing of archived MicroRNAs from up to 35-year-old clinical FFPE specimens. Int J Mol Sci.

[49] Loudig O, Liu C, Rohan T, Ben-Dov IZ (2018). Retrospective MicroRNA sequencing: complementary DNA library preparation protocol using formalin-fixed paraffin-embedded RNA specimens. J Vis Exp JoVE.

[50] Kotorashvili A, Ramnauth A, Liu C, Lin J, Ye K, Kim R, Hazan R, Rohan T, Fineberg S, Loudig O (2012). Effective DNA/RNA co-extraction for analysis of microRNAs, mRNAs, and genomic DNA from formalin-fixed paraffin-embedded specimens. PLoS One.

[51] Mazeh H, Deutch T, Karas A, Bogardus KA, Mizrahi I, Gur-Wahnon D, Ben-Dov IZ (2018). Next-generation sequencing identifies a highly accurate miRNA Panel that distinguishes well-differentiated thyroid cancer from benign thyroid nodules. Cancer Epidemiol Biomarkers Prev.

[52] Ryser MD, Hendrix LH, Worni M, Liu Y, Hyslop T, Hwang ES (2019). Incidence of ductal carcinoma in situ in the United States, 2000–2014. Cancer Epidemiol Biomark Prev.

[53] Sanders ME, Schuyler PA, Dupont WD, Page DL (2005). The natural history of low-grade ductal carcinoma in situ of the breast in women treated by biopsy only revealed over 30 years of long-term follow-up. Cancer.

[54] Bremer T, Whitworth PW, Patel R, Savala J, Barry T, Lyle S, Leesman G, Linke SP, Jirström K, Zhou W, Amini RM, Wärnberg F (2018). A biological signature for breast ductal carcinoma *in situ* to predict radiotherapy benefit and assess recurrence risk. Clin Cancer Res.

[55] Bergholtz H, Lien TG, Swanson DM (2020). Contrasting DCIS and invasive breast cancer by subtype suggests basal-like DCIS as distinct lesions. npj Breast Cancer.

[56] Siow ZR, De Boer RH, Lindeman GJ, Mann GB (2018). Spotlight on the utility of the oncotype DX® breast cancer assay. Int J Womens Health.

[57] Valeri N, Braconi C, Gasparini P, Murgia C, Lampis A, Paulus-Hock V, Hart JR, Ueno L, Grivennikov SI, Lovat F, Paone A, Cascione L, Sumani KM, Veronese A, Fabbri M, Carasi S, Alder H, Lanza G, Gafa' R, Moyer MP, Ridgway RA, Cordero J, Nuovo GJ, Frankel WL, Rugge M, Fassan M, Groden J, Vogt PK, Karin M, Sansom OJ, Croce CM (2014). MicroRNA-135b promotes cancer progression by acting as a downstream effector of oncogenic pathways in colon cancer. Cancer Cell.

[58] Zhang YJ, Ma YS, Xia Q, Yu F, Lv ZW, Jia CY, Jiang XX, Zhang L, Shao YC, Xie WT, Lu GX, Yv XQ, Zhong P, Fu D, Wang XF (2018). MicroRNA-mRNA integrated analysis based on a case of well-differentiated thyroid cancer with both metastasis and metastatic recurrence. Oncol Rep.

[59] Zuberi M, Mir R, Khan I, Javid J, Guru SA, Bhat M, Sumi MP, Ahmad I, Masroor M, Yadav P, Vishnubhatla S, Saxena A (2020). The promising signatures of circulating microRNA-145 in epithelial ovarian cancer patients. Microrna.

[60] Zou MX, Huang W, Wang XB, Lv GH, Li J, Deng YW (2014). Identification of miR-140-3p as a marker associated with poor prognosis in spinal chordoma. Int J Clin Exp Pathol.

[61] Halvorsen AR, Helland Å, Gromov P, Wielenga VT, Talman MM, Brunner N, Sandhu V, Børresen-Dale AL, Gromova I, Haakensen VD (2017). Profiling of microRNAs in tumor interstitial fluid of breast tumors - a novel resource to identify biomarkers for prognostic classification and detection of cancer. Mol Oncol.

[62] Quan Y, Huang X, Quan X (2018). Expression of miRNA-206 and miRNA-145 in breast cancer and correlation with prognosis. Oncol Lett.

[63] Zhou X, Lu Z, Wang T, Huang Z, Zhu W, Miao Y (2018). Plasma miRNAs in diagnosis and prognosis of pancreatic cancer: a miRNA expression analysis. Gene.

[64] Zhang YH, Jin M, Li J, Kong X (2020). Identifying circulating miRNA biomarkers for early diagnosis and monitoring of lung cancer. Biochim Biophys Acta Mol Basis Dis.

[65] Cao Z, Qiu J, Yang G, Liu Y, Luo W, You L, Zheng L, Zhang T (2020). MiR-135a biogenesis and regulation in malignancy: a new hope for cancer research and therapy. Cancer Biol Med.

[66] Chen L, Li Y, Fu Y, Peng J, Mo MH, Stamatakos M, Teal CB, Brem RF, Stojadinovic A, Grinkemeyer M, McCaffrey TA, Man YG, Fu SW (2013). Role of deregulated microRNAs in breast cancer progression using FFPE tissue. PLoS One.

[67] Qi L, Bart J, Tan LP, Platteel I, Sluis TV, Huitema S, Harms G, Fu L, Hollema H, Berg AV (2009). Expression of miR-21 and its targets (PTEN, PDCD4, TM1) in flat epithelial atypia of the breast in relation to ductal carcinoma in situ and invasive carcinoma. BMC Cancer.

[68] Li S, Pu T, Xiao L, Gao H, Li L, Ye F, Liu Y, Bu H (2019). Screening of recurrence related MicroRNA in ductal carcinoma *in situ* and functional study of MicroRNA-654-5p. J Breast Cancer.

[69] Hannafon BN, Sebastiani P, de las Morenas A, Lu J, Rosenberg CL (2011). Expression of microRNA and their gene targets are dysregulated in preinvasive breast cancer. Breast Cancer Res BCR.

[70] Sempere LF, Christensen M, Silahtaroglu A, Bak M, Heath CV, Schwartz G, Wells W, Kauppinen S, Cole CN (2007). Altered MicroRNA expression confined to specific epithelial cell subpopulations in breast cancer. Can Res.

[71] Cizeron-Clairac G, Lallemand F, Vacher S, Lidereau R, Bieche I, Callens C (2015). MiR-190b, the highest up-regulated miRNA in ERα-positive compared to ERα-negative breast tumors, a new biomarker in breast cancers?. BMC Cancer.

[72] Rodríguez-González FG, Sieuwerts AM, Smid M, Look MP, Meijer-van Gelder ME, de Weerd V, Sleijfer S, Martens JW, Foekens JA (2011). MicroRNA-30c expression level is an independent predictor of clinical benefit of endocrine therapy in advanced estrogen receptor positive breast cancer. Breast Cancer Res Treat.

[73] Rohan TE, Wang T, Weinmann S, Wang Y, Lin J, Ginsberg M, Loudig O (2019). A miRNA expression signature in breast tumor tissue is associated with risk of distant metastasis. Cancer Res.

[74] Zhang P, Wang L, Rodriguez-Aguayo C, Yuan Y, Debeb BG, Chen D, Sun Y, You MJ, Liu Y, Dean DC, Woodward WA, Liang H, Yang X, Lopez-Berestein G, Sood AK, Hu Y, Ang KK, Chen J, Ma L (2014). miR-205 acts as a tumour radiosensitizer by targeting ZEB1 and Ubc13. Nat Commun.

[75] Vislovukh A, Kratassiouk G, Porto E, Gralievska N, Beldiman C, Pinna G, El'skaya A, Harel-Bellan A, Negrutskii B, Groisman I (2013). Proto-oncogenic isoform A2 of eukaryotic translation elongation factor eEF1 is a target of miR-663 and miR-744. Br J Cancer.

[76] Tribollet V, Barenton B, Kroiss A, Vincent S, Zhang L, Forcet C, Cerutti C, Périan S, Allioli N, Samarut J, Vanacker JM (2016). miR-135a inhibits the invasion of cancer cells via suppression of ERRα. PLoS One.

[77] Jiang D, Zhou B, Xiong Y, Cai H (2019). miR-135 regulated breast cancer proliferation and epithelial-mesenchymal transition acts by the Wnt/β-catenin signaling pathway. Int J Mol Med.

[78] Chen X, Lu P, Wang DD, Yang SJ, Wu Y, Shen HY, Zhong SL, Zhao JH, Tang JH (2016). The role of miRNAs in drug resistance and prognosis of breast cancer formalin-fixed paraffin-embedded tissues. Gene.

[79] Piovan C, Palmieri D, Di Leva G, Braccioli L, Casalini P, Nuovo G, Tortoreto M, Sasso M, Plantamura I, Triulzi T, Taccioli C, Tagliabue E, Iorio MV, Croce CM (2012). Oncosuppressive role of p53-induced miR-205 in triple negative breast cancer. Mol Oncol.

[80] Iorio MV, Casalini P, Piovan C, Di Leva G, Merlo A, Triulzi T, Ménard S, Croce CM, Tagliabue E (2009). microRNA-205 regulates HER3 in human breast cancer. Can Res.

[81] Gregory PA, Bert AG, Paterson EL, Barry SC, Tsykin A, Farshid G, Vadas MA, Khew-Goodall Y, Goodall GJ (2008). The miR-200 family and miR-205 regulate epithelial to mesenchymal transition by targeting ZEB1 and SIP1. Nat Cell Biol.

