# Supplementary material for: MiRNA expression deregulation correlates with the Oncotype DX® DCIS score
Source: Breast Cancer Res. 2022 Sep 12;24:62. doi: 10.1186/s13058-022-01558-4 (PMC9469592; doi:10.1186/s13058-022-01558-4)
Supplement: Supplementary file 1 — Additional file 1. Fig. 1: Correlation between nuclear grade of the DCIS specimens and the Oncotype DX® DCIS risk score or the miRNA composite score. a. Result tables displaying distribution of the nuclear grades of 40 out of the 41 DCIS samples (One Low Oncotype DX® DCIS risk score sample was missing nuclear grade) by Oncotype DX® DCIS risk scores. The left table displays the distribution of the nuclear grades of the DCIS samples between the three Oncotype DX® DCIS risk score groups (i.e., low-, intermediate-, and high-risk scores), providing a nonsignificant p value of 0.07906. The right table displays distribution of the nuclear grade of the DCIS samples between the low- and the intermediate-/high-risk score groups, which provides a significant p value of 0.01525. b. correlation plot between the DCIS sample’s Oncotype DX® DCIS risk scores and the three nuclear grades (Pearson correlation coefficient (Rho) r = 0.506). c. Correlation plot between the DCIS sample’s miRNA composite risk scores and the three nuclear grades (Pearson correlation coefficient (Rho) r = 0.168). [file 13058_2022_1558_MOESM1_ESM.pdf]

a.

Nuclear Grade (N)	Oncotype DX® DCIS Risk Score Group		
	Low *	Intermediate	High
N1 - Low	6	0	0
N2 - Intermediate	14	4	2
N3 - High	5	6	3
Pearson Chi-squared test<br>X-squared =8.3657 , degree of freedom (df) = 4, p-value = 0.07906			

* Total of 26 Low Oncotype DX® DCIS risk patients, with only 25 known nuclear grades

Nuclear Grade (N)	Oncotype DX® DCIS Risk Score Group	
	Low *	Intermediate/High
N1 - Low	6	0
N2 - Intermediate	14	6
N3 - High	5	9
Pearson Chi-squared test<br>X-squared =8.3657 , degree of freedom (df) = 2, p-value = 0.01525		
Fisher's Exact Test for count data<br>p-value = 0.01648 , alternative hypothesis: two.sided		

b.

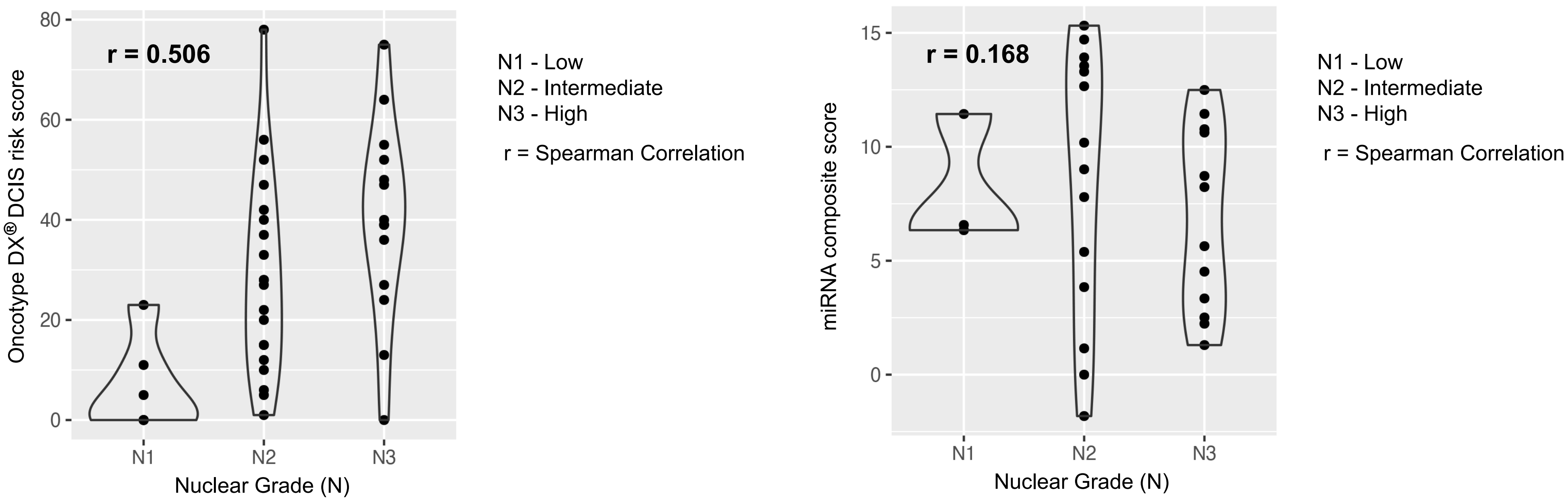

Additional Figure. 1
